# A tumor microenvironment model of chronic lymphocytic leukemia enables drug sensitivity testing to guide precision medicine

**DOI:** 10.1038/s41420-023-01426-w

**Published:** 2023-04-13

**Authors:** Johanne U. Hermansen, Yanping Yin, Aleksandra Urban, Camilla V. Myklebust, Linda Karlsen, Katrine Melvold, Anders A. Tveita, Kjetil Taskén, Ludvig A. Munthe, Geir E. Tjønnfjord, Sigrid S. Skånland

**Affiliations:** 1 Department of Cancer Immunology, Institute for Cancer Research, Oslo University Hospital, Oslo, Norway; 2 K. G. Jebsen Centre for B Cell Malignancies, Institute of Clinical Medicine, University of Oslo, Oslo, Norway; 3 Department of Haematology, Oslo University Hospital, Oslo, Norway; 4 Department of Immunology, Oslo University Hospital, Oslo, Norway

**Keywords:** Chronic lymphocytic leukaemia, Cell growth

## Abstract

The microenvironment of chronic lymphocytic leukemia (CLL) cells in lymph nodes, spleen, and bone marrow provides survival, proliferation, and drug resistance signals. Therapies need to be effective in these compartments, and pre-clinical models of CLL that are used to test drug sensitivity must mimic the tumor microenvironment to reflect clinical responses. Ex vivo models have been developed that capture individual or multiple aspects of the CLL microenvironment, but they are not necessarily compatible with high-throughput drug screens. Here, we report on a model that has reasonable associated costs, can be handled in a regularly equipped cell lab, and is compatible with ex vivo functional assays including drug sensitivity screens. The CLL cells are cultured with fibroblasts that express the ligands APRIL, BAFF and CD40L for 24 h. The transient co-culture was shown to support survival of primary CLL cells for at least 13 days, and mimic in vivo drug resistance signals. Ex vivo sensitivity and resistance to the Bcl-2 antagonist venetoclax correlated with in vivo responses. The assay was used to identify treatment vulnerabilities and guide precision medicine for a patient with relapsed CLL. Taken together, the presented CLL microenvironment model enables clinical implementation of functional precision medicine in CLL.

## Introduction

Targeted therapies have dramatically improved the management of chronic lymphocytic leukemia (CLL). However, patients who progress after sequential lines of therapy are faced with a dismal prognosis [1, 2]. A better understanding of the molecular alterations that lead to disease progression, as well as identification of novel treatment options in relapsed disease, may contribute to optimized treatment schedules.

Functional precision medicine is based on the principle to test the sensitivity to different drugs directly on the patient’s tumor cells [3, 4]. This approach to guide treatment decisions has proven valuable in relapsed/refractory acute leukemia and lymphoma [5, 6]. While large drug-sensitivity screens have been performed on CLL cells as well [7, 8], this has not yet resulted in systematic clinical validation of the method to guide precision medicine in this disease. An obvious challenge with screening primary CLL cells is that they undergo rapid, spontaneous apoptosis outside of their natural tumor microenvironment. It is therefore necessary to model the tumor microenvironment to support cell viability during the course of the experiment so that only drug-induced cell death is assessed. Furthermore, the tumor microenvironment contributes to drug resistance signals [9]. These signals should therefore be present during the drug testing so that the measured ex vivo drug sensitivity reflects the in vivo sensitivity to any given treatment.

The CLL microenvironment is composed of a variety of cell types that provide proliferation and survival signals to the tumor cells [10]. Nurse-like cells (monocytes or macrophages) secrete chemokines and cytokines such as APRIL and BAFF which promote anti-apoptotic signals [11]. T cells, on the other hand, interact with CLL cells via the CD40L/CD40 axis, and this leads to the induction of cell signals that overlap with B cell receptor induced signaling. These signals regulate apoptosis via modified expression of pro-survival proteins including Bcl-xL and Mcl-1 [12]. A number of culturing protocols have been developed which take into consideration such microenvironmental stimuli and/or the spatial organization of the microenvironment [13–17]. Some of these protocols suggest the addition of soluble cytokines to the growth medium, which can be costly for large-scale screening setups. Protocols that require 3D modeling of the tumor microenvironment, on the other hand, may be less accessible to the non-expert researcher.

Here, we aimed to develop a standardized microenvironment model that is compatible with ex vivo functional assays including drug sensitivity screens; has reasonable associated costs; and can be handled by the average researcher in a regularly equipped cell lab. We did not wish to rely on bone marrow stromal cells as this would be invasive to the patients, introduce heterogeneity between experiments, and possibly reduce the throughput of the screens due to limited material available. With these considerations in mind, we engineered fibroblasts that overexpress the key microenvironment factors APRIL, BAFF and CD40L. Although this model reflects only some of the factors of the CLL microenvironment, co-culture of primary CLL cells with these fibroblasts provided anti-apoptotic signals and prevented induction of spontaneous apoptosis. Importantly, a transient co-culture was sufficient to support prolonged CLL survival. This means that no fibroblasts need to be present during subsequent functional analyses. Any noise from the supporting cells could therefore be excluded. By performing a case study of a relapsed CLL patient, we show that ex vivo sensitivity to the Bcl-2 antagonist venetoclax correlates with in vivo response to the same treatment. Furthermore, the drug sensitivity testing identified treatment vulnerabilities which were used to guide precision medicine for the patient. Taken together, our findings suggest that the CLL tumor microenvironment model is compatible with ex vivo drug sensitivity testing to guide treatment decisions, opening for the possibility of clinical implementation of functional precision medicine in CLL.

## Results

### An ex vivo model of the CLL tumor microenvironment

**Fig. 1:**
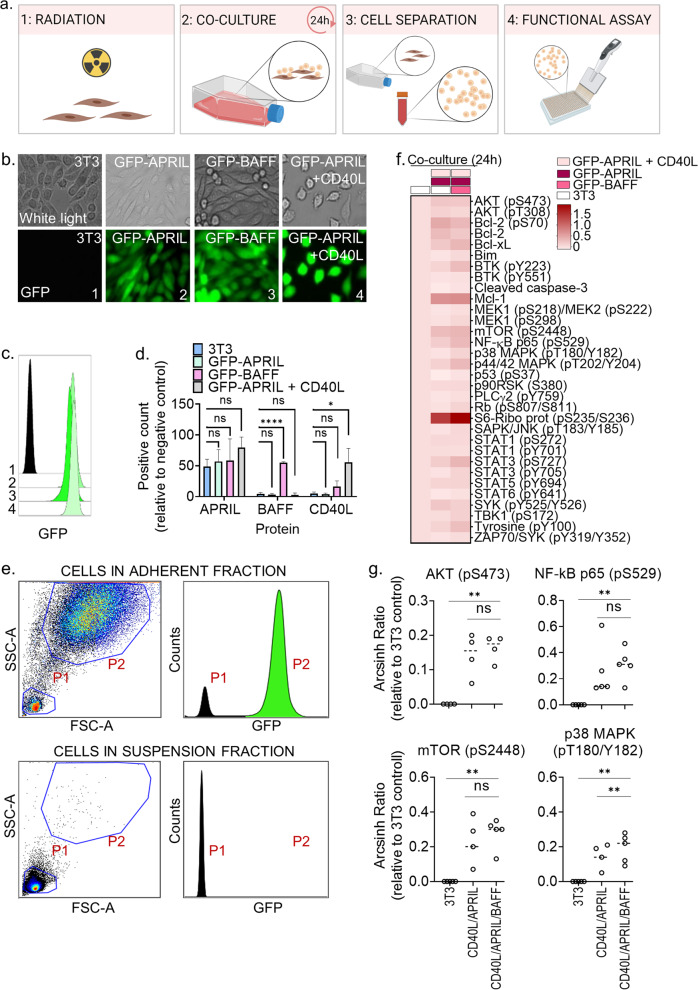
Co-culture with CD40L/APRIL/BAFF expressing fibroblasts stimulates CLL signaling. **a** Schematic illustration of the co-culture protocol. The figure was made with BioRender.com. **b** Fibroblasts were transduced with green fluorescent protein (GFP)-APRIL, GFP-BAFF or GFP-APRIL + CD40L. Expression of GFP was analyzed with an Axio Vert.A1 fluorescence microscope from Zeiss (Oberkochen, Germany; lower panels). The upper panels show the phase contrast. **c** Wild-type fibroblasts (1) and fibroblasts transduced with GFP-APRIL (2), GFP-BAFF (3) or GFP-APRIL + CD40L (4) were fixed and analyzed with a BD LSR Fortessa flow cytometer. The data were analyzed in Cytobank (https://cellmass.cytobank.org/cytobank/). The histogram shows relative GFP signal in the different cell lines. **d** Expression of APRIL, BAFF, and CD40L in wild-type fibroblasts (blue bars) and fibroblasts transduced with GFP-APRIL (green bars), GFP-BAFF (pink bars), or GFP-APRIL + CD40L (gray bars) were analyzed by flow cytometry. The cells were stained with the corresponding antibodies and gated for single cells. Signals are shown as percentage of positive cells relative to background signal from secondary antibody alone (mean ± standard deviation (SD), *n* = 2) Statistics were performed with a one-way ANOVA with multiple comparisons. *p < 0.05, ****p < 0.0001. **e** Peripheral blood mononuclear cells (PBMCs) from CLL patient samples were co-cultured at a 10:1 ratio with irradiated wild-type fibroblasts, GFP-APRIL and GFP-APRIL + CD40L fibroblasts (ratio 1:1:1) for 24 h. The CLL cells were then separated from the adherent fibroblast layer by carefully re-suspending and transferring the cell medium to a separate container. The remaining fibroblast layer was detached by trypsination. The two cell fractions were fixed separately and analyzed by flow cytomtery for detection of cell size, granularity, and GFP signals. The upper panels show signals in the adherent cell fraction, while the lower panels show signals in the soluble cell fraction. Size and granularity of the cells were determined by plotting side scatter (SSC-A) against forward scatter (FSC-A; left panels). The GFP signals in the gated populations (P1 and P2) are shown as counts (right panels). **f** Primary CLL cells were co-cultured with wild-type 3T3 fibroblasts only (left column), 3T3, GFP-APRIL, and GFP-APRIL + CD40L fibroblasts (middle column), or GFP-BAFF, GFP-APRIL, and GFP-APRIL + CD40L fibroblasts (right column) for 24 h. The cells were then fixed, permeabilized and stained with phospho-antibodies. The samples were analyzed with a BD LSR Fortessa flow cytometer. Signals are shown as median relative to the unstimulated wild-type control (first row) as arcsinh ratio. **g** The experiments were performed as described in f) on 4-5 CLL patient samples, including the patient sample shown in (**f**). **p < 0.01 calculated by a two-tailed paired *t* test comparing 3T3 fibroblasts (3T3) to GFP-APRIL + CD40L (CD40L/APRIL), and GFP-APRIL + CD40L (CD40L/APRIL) to GFP-BAFF and GFP-APRIL + CD40L (CD40L/APRIL/BAFF) assuming Gaussian distribution.

To make a model of the CLL tumor microenvironment that supports prolonged survival of the CLL cells, we studied the effect of culturing CLL cells with the ligands APRIL, BAFF, and CD40L—key stimuli provided by nursing cells and T cells in the tumor microenvironment [10]. For the model to be cost-effective and compatible with large-scale drug sensitivity screens, NIH/3T3 fibroblast cell lines stably expressing GFP-APRIL or GFP-BAFF, and fibroblast L cells co-expressing GFP-APRIL + CD40L were generated. Expression of GFP was confirmed by fluorescence microscopy (Fig. 1b) and flow cytometry (Fig. 1c). The morphology of the 3T3 fibroblasts and L fibroblasts was observed to be somewhat different (Fig. 1b). Ligand expression of APRIL, BAFF, and CD40L was confirmed by flow cytometry (Fig. 1d). APRIL was endogenously expressed in the 3T3 control fibroblasts (Fig. 1d), but the GFP-APRIL transfected fibroblasts showed additional, although statistically insignificant, APRIL expression (Fig. 1d, green and gray bars). This made it difficult to assess the independent contribution of APRIL stimulation on CLL cells. However, soluble APRIL was previously shown to protect CLL cells from spontaneous apoptosis [18]. BAFF and CD40L were only expressed in the GFP-BAFF and GFP-APRIL + CD40L transfected cell lines, respectively (Fig. 1d).

To test the functional effects of the tumor microenvironment model, peripheral blood mononuclear cells from CLL patients (PBMCs; here referred to as CLL cells) were co-cultured with fibroblasts expressing GFP-APRIL, GFP-BAFF and GFP-APRIL + CD40L for 24 h. The fibroblasts were irradiated prior to the co-culture to prevent further proliferation. The CLL cells were then separated from the adherent fibroblast layer by pouring off the cell medium containing the CLL cells. Complete separation of the CLL cells from the adherent fibroblast layer was confirmed by flow cytometry by distinction of cell size, and the presence or absence of GFP signal, in the suspension fraction (P1; CLL cells) and the adherent fraction (P2; fibroblasts) of the co-culture (Fig. 1e).

High-throughput protein profiling showed that co-culture with GFP-APRIL + CD40L fibroblasts for 24 h significantly induced CLL signaling in several pathways related to cell proliferation, including AKT (pS473), mTOR (pS2448), NF-κB p65 (pS529), and p38 MAPK (pT180/Y182) (Fig. 1f, g). This is in line with previous findings indicating that activation of CD40 leads to activation of PI3K/AKT, p44/42 MAPK, JAK/STAT and NF-κB [12, 19]. Co-culture of CLL cells with the combination of GFP-APRIL, GFP-BAFF and GFP-APRIL + CD40L fibroblasts for 24 h resulted in a significant additive effect on the phosphorylation signals of p38 MAPK (pT180/Y182) relative to co-culture with GFP-APRIL + CD40L fibroblasts only (Fig. 1g). APRIL and BAFF bind to B cell maturation antigen (BCMA) which is involved in activation of NF-κB and p38 MAPK, among others [18, 20]. This may explain the observed additive effect of BAFF stimulation on p38 MAPK (pT180/Y182) activation. Other stimulation-induced effects on cell signaling were not significantly different between the GFP-APRIL + CD40L and the GFP-APRIL, GFP-BAFF and GFP-APRIL + CD40L conditions (Fig. 1f, g).

### Transient CD40L/APRIL/BAFF stimulation of primary CLL cells induces durable anti-apoptotic and drug resistance signals

**Fig. 2:**
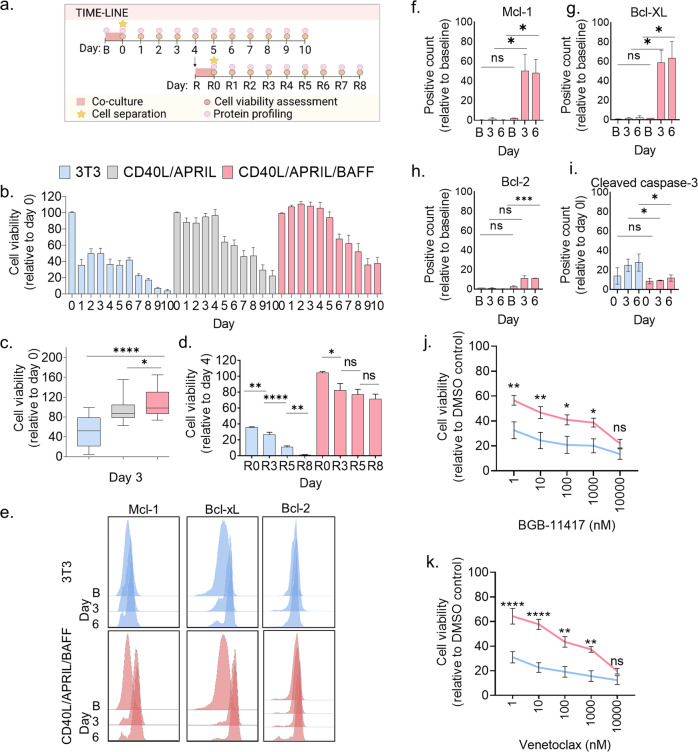
Transient CD40L/APRIL/BAFF stimulation of primary CLL cells induces durable anti-apoptotic and drug resistance signals. **a** Illustration of the protocol setup. Co-culture and separation of the CLL cells from the fibroblasts are indicated in the time-lines. A baseline (B) CLL sample was collected for protein profiling before the co-culture was started (upper time-line). The co-culture was performed for 24 h (pink square) before the CLL cells were separated from the fibroblast layer (star). The CLL cells were kept in culture and collected at days 0–10, as indicated. The cells were assessed both for protein profiling (light pink circles) and cell viability (dark pink circles). At day 4, part of the CLL cells were exposed to a 24 h restimulation (R) with fibroblasts (lower time-line). The CLL cells were then separated from the fibroblasts (star) and kept in culture. CLL samples were collected for protein profiling (light pink circles) and cell viability assessment (dark pink circles) at the indicated time-points R0–R8. The figure was made with BioRender.com. **b** Peripheral blood mononuclear cells (PBMCs) from CLL patient samples were co-cultured at a 10:1 ratio with irradiated wild-type 3T3 fibroblasts; 3T3, GFP-APRIL and GFP-APRIL + CD40L fibroblasts (ratio 1:1:1); or GFP-APRIL, GFP-BAFF and GFP-APRIL + CD40L fibroblasts (ratio 1:1:1) for 24 h. The CLL cells were then separated from the adherent fibroblast layer and transferred to 384-well plates (10,000 cells/25 µl). Cell viability was assessed with the CellTiter-Glo luminescent cell viability assay for 10 consecutive days. The signals were normalized to the signal at day 0 (mean ± standard error of the mean (SEM), *n* = 5). **c** The experiments are described in (**b**). The CellTiter-Glo signals detected at day 3 are shown in a box plot (min to max, *n* = 5). Statistics were performed with a two-tailed unpaired *t* test. *p < 0.05, ****p < 0.0001. **d** PBMCs from CLL patient samples were co-cultured with the indicated fibroblast cell lines for 24 h as described in (**a**). After cell separation, the PBMCs were kept in culture. On day 4, the PBMCs were exposed to the same co-culture (re-stimulation) for another 24 h. Cell viability was assessed with the CellTiter-Glo luminescent cell viability assay at the indicated days. The signals were normalized to the cell viability detected at day 4 in the original setup. (R0; mean ± standard error of the mean (SEM), *n* = 3). Statistics were performed with an unpaired *t* test assuming both populations have the same SEM. *p < 0.05, **p < 0.01, ****p < 0.0001. **e** PBMCs from the same CLL patient samples were either fixed at baseline (B) or after co-culture with the different fibroblast lines for 24 h as described in (**a**). The cells were then permeabilized and stained with the indicated antibodies. Signals were analyzed by flow cytometry. The histograms show one representative experiment of 3 experiments. **f**–**i** The experiments are described in (**e**). The first 3 bars show 3T3 fibroblast co-cultured CLL cells. The 3 bars to the right show CD40L/APRIL/BAFF co-cultured CLL cells. The CLL cells from (**e**) were analyzed as percent positive counts relative to the baseline (mean ± SEM, *n* = 3). The graphs show measurements for baseline (B) or day 0, and day 3 and day 6 after co-culture. Statistics were performed with a two-tailed unpaired *t* test. *p < 0.05. **j**, **k**. PBMCs from CLL patient samples were co-cultured with 3T3 fibroblasts (blue line) or CD40L/APRIL/BAFF fibroblasts (pink line) for 24 h as described in (**a**). The CLL cells were separated from the adherent fibroblast layer and treated with BGB-11417 (**j**) or venetoclax (**k**) (1–10,000 nM) for 72 h in a 384-well plate (10,000 cells/25 µl). Cell viability was assessed with the CellTiter-Glo luminescent cell viability assay. Signals were normalized to the negative (0.1% DMSO) control (mean ± SEM, *n* = 6). Statistics were performed with a two-tailed 2way ANOVA with Bonferroni corrections. *p < 0.05, **p < 0.01, ****p < 0.0001.

To study the durability of the co-culture-induced signals, we separated the CLL cells from the fibroblasts after 24 h and analyzed cell viability and cell signaling in the CLL cells for the next 10 days (Fig. 2a). To also examine the contributions of APRIL, BAFF, and CD40L on CLL cell survival, we performed the 10-day CellTiter-Glo luminescent viability assay following 24 h co-culture with either control fibroblasts, GFP-APRIL + CD40L fibroblasts or GFP-APRIL, GFP-BAFF, and GFP-APRIL + CD40L fibroblasts (Fig. 2a, b). Up to 40% of the CD40L/APRIL/BAFF stimulated CLL cells were viable 10 days post co-culture (Fig. 2b), while only 4% of the CLL cells from the control co-culture were alive at the same time (Fig. 2b). The CLL cell survival was also significantly higher in the CD40L/APRIL/BAFF co-culture than in the APRIL/CD40L co-culture at day 3 (Fig. 2c). This condition was therefore used in further experiments.

To investigate if the stimulation-supported survival of the CLL cells could be further prolonged, a 24 h co-culture was repeated at day 4 of the time-course experiment (Fig. 2a, d). The fraction of viable CLL cells from the CD40L/APRIL/BAFF co-culture at day R8 (13 days after the primary co-culture) was significantly higher compared to the fraction of viable CLL cells from the control fibroblast co-culture (70% vs 1%) (Fig. 2d). This demonstrates that the model supports prolonged survival of primary CLL cells.

To substantiate the observed cell viability data, expression level of anti-apoptotic markers was investigated by flow cytometry. Mcl-1, Bcl-xL, and Bcl-2 were significantly upregulated in CLL cells in response to CD40L/APRIL/BAFF co-culture, but not in response to control co-culture (Fig. 2e–h). In agreement with this, we observed increased cleavage of caspase-3 in CLL cells (Fig. 2i), as well as a higher proportion of dead cells over time (Supplementary Fig. 1a), in the control co-culture than in the CD40L/APRIL/BAFF co-culture. This is in agreement with previous reports of CD40L-induced modulation of pro-survival protein expression [21, 22]. BAFF activates STAT3 and enhances transcription of the anti-apoptotic protein Mcl-1 [23]. This may indicate that the observed additional increased signaling of STAT3 (pS737) (Fig. 1f) and upregulation of Mcl-1 (Fig. 2e, f) are driven by BAFF signaling. Continuous stimulation of CLL cells with CD40L in combination with other cytokines over several days has been reported to induce CLL cell proliferation [16, 17]. While we observed increased expression of the proliferation markers Ki-67 and PCNA in response to the CD40L/APRIL/BAFF co-culture (Supplementary Fig. 1b), we did not detect cell proliferation in a CFSE proliferation assay (data not shown). This may be due to the limited stimulation time (24 h) with CD40L/APRIL/BAFF.

It has been reported that CD40L induces resistance to the Bcl-2 antagonist venetoclax [24]. To test the CLL sensitivity to the Bcl-2 antagonists BGB-11417 and venetoclax after CD40L/APRIL/BAFF stimulation, we treated unstimulated or stimulated CLL cells with physiologically relevant concentrations (1–10,000 nM) of BGB-11417 or venetoclax for 24 h, a time-point at which the viability of unstimulated CLL cells was less affected by spontaneous apoptosis (see Fig. 2b). As expected, we observed that unstimulated CLL cells were highly sensitive to the drugs (Fig. 2j, k). After treatment with 10 nM of either BGB-11417 or venetoclax, only 25% of the cells remained viable (Fig. 2j, k, blue lines). However, for the CD40L/APRIL/BAFF stimulated CLL cells, we observed approximately 50% viability of CLL cells after treatment with 10 nM BGB-11417 and 60% viability after treatment with 10 nM venetoclax (Fig. 2j, k, pink lines). The cell viability was significantly higher for each concentration (except 10,000 nM) of BGB-11417 or venetoclax for CLL cells co-cultured with CD40L/APRIL/BAFF fibroblasts compared to CLL cells co-cultured with control fibroblasts (Fig. 2j, k). These findings support the notion that microenvironmental factors contribute to drug resistance in CLL cells.

### Ex vivo drug sensitivity correlates with in vivo sensitivity in a CLL patient case and guides precision medicine

**Fig. 3:**
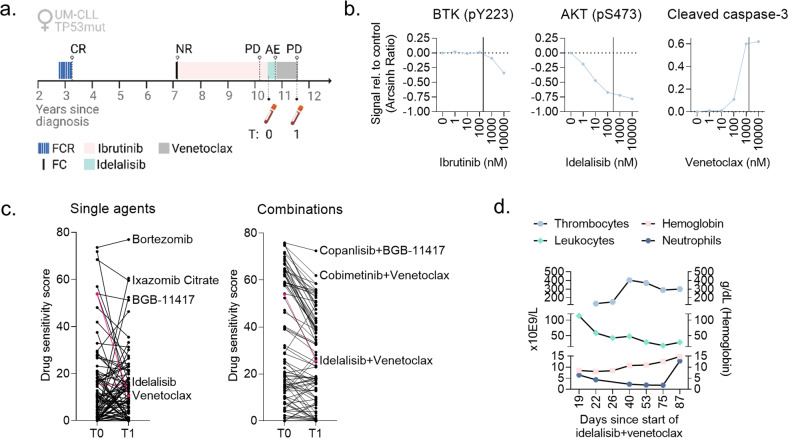
Ex vivo drug sensitivity correlates with in vivo sensitivity in a CLL patient case and guides precision medicine. **a** Time-line illustrating the treatment history of the CLL patient case. T0 and T1 indicate time-points when blood samples were collected from the patient. AE, adverse event; CR, complete response; FC(R), fludarabine + cyclophosphamide (+ rituximab); NR, no response; PD, progressive disease. The figure was created with BioRender.com. **b** Peripheral blood mononuclear cells (PBMCs) from sample T0 were treated with the indicated drug (ibrutinib, idelalisib or venetoclax) at five different concentrations (1–10,000 nM) for 30 min, followed by stimulation with anti-IgM (10 μg/ml) for 5 min. The cells were then fixed, permeabilized and stained with antibodies against CD19 and intracellular proteins as indicated. The cells were analyzed with a BD LSR Fortessa flow cytometer, and the data were analyzed with Cytobank (https://cellmass.cytobank.org/cytobank/). The graphs show the median signals as Arcsinh Ratio relative to DMSO (0.1%) control treated cells for the indicated proteins in CD19^+^ B cells. The vertical lines indicate the reported peak plasma concentration for each drug. **c** PBMCs from samples T0 and T1 were co-cultured with CD40L/APRIL/BAFF fibroblasts for 24 h to prevent spontaneous apoptosis of the CLL cells. The CLL cells were then separated from the fibroblast layer and treated with a drug library consisting of 94 single agents (left) and 87 drug combinations (right) at 5 different concentrations (1–10,000 nM; 0.1–1000 nM for copanlisib and dasatinib) for 72 h. Cell viability was then assessed with the CellTiter-Glo luminescent cell viability assay. The graphs show the drug sensitivity scores to the different treatments, which were calculated based on the area under the concentration-response curve. High score indicates high sensitivity to the treatment. Select treatments are annotated, and the responses to idelalisib, venetoclax, and idelalisib + venetoclax are indicated in pink. **d** Blood counts of the CLL patient in response to treatment with idelalisib + venetoclax combination. Note the non-linear time scale. Graphs were made with GraphPad Prism 9 (San Diego, CA, USA).

**Fig. 4:**
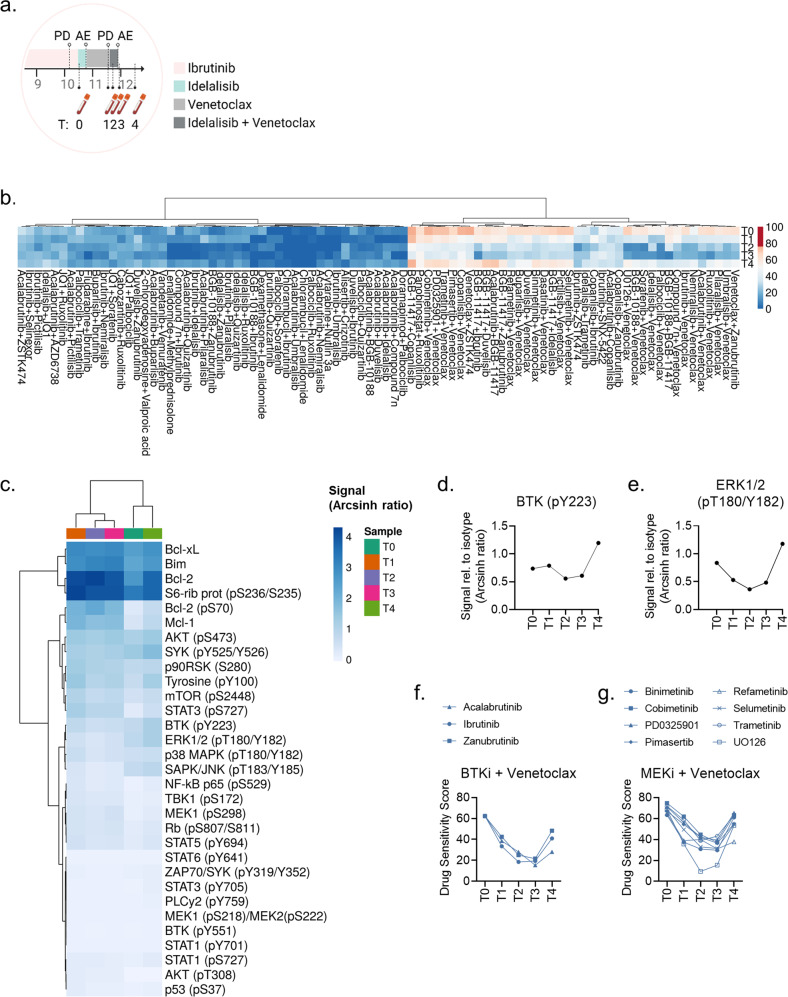
Evolution of ex vivo drug sensitivity in response to sequential therapies and treatment stop. **a** Cropped time-line illustrating the treatment sequence for the CLL patient following ibrutinib therapy. T0-T4 indicate time-points when blood samples were collected from the patient. AE, adverse event; PD, progressive disease. The figure was created with BioRender.com. **b** PBMCs from T0-T4 were co-cultured with CD40L/APRIL/BAFF fibroblasts for 24 h to prevent spontaneous apoptosis of the CLL cells. The CLL cells were then separated from the fibroblast layer and treated with the indicated drug combinations for 72 h. Cell viability was assessed with the CellTiter-Glo luminescent cell viability assay. The drug sensitivity score was calculated for each treatment based on the area under the concentration-response curve. A high score indicates high sensitivity to the treatment. The heatmap was created using ClustVis (https://biit.cs.us.ee/clustvis/). **c** Freshly thawed peripheral blood mononuclear cells (PBMCs) from T0-T4 were fixed, permeabilized and stained with antibodies against the indicated proteins (rows). Signals were detected in CD19^+^ B cells by flow cytometry. Raw data were transformed to an arcsinh ratio relative to the signal of an isotype control, which was set to zero. The heatmap was created using ClustVis (https://biit.cs.us.ee/clustvis/). Both rows and columns were clustered using Manhattan distance and Ward linkage algorithm. **d**, **e** Phosphorylation levels of BTK (pY223) and ERK1/2 (pT180/Y182) are shown for experiments described in (**c**). **f**, **g** PBMCs from T0-T4 were co-cultured with CD40L/APRIL/BAFF fibroblasts for 24 h to prevent spontaneous apoptosis of the CLL cells. The CLL cells were then separated from the fibroblast layer and treated with the indicated drug combinations for 72 h. Cell viability was assessed with the CellTiter-Glo luminescent cell viability assay. The drug sensitivity score was calculated for each treatment based on the area under the concentration-response curve. A high score indicates high sensitivity to the treatment.

To study whether ex vivo drug sensitivity can predict in vivo response to the same treatment, we studied a CLL patient case. A 64-year-old female patient presented with unmutated IGVH (UM-CLL), *TP53* mutation, and refractory disease after sequential treatment with FCR (fludarabine, cyclophosphamide, rituximab), FC, and ibrutinib (Fig. 3a). PBMCs were isolated from blood samples collected at the indicated time-points (Figs. 3a, 4a), and cryopreserved as previously described [25].

To study the sensitivity to the targeted therapies ibrutinib, idelalisib and venetoclax in CLL cells collected at time T0 (after progression on ibrutinib, Fig. 3a), the cells were treated with each drug at clinically relevant drug concentrations (1–10,000 nM) for 30 min, followed by 5 min anti-IgM stimulation. The drug concentration corresponding to the reported peak plasma concentration for each agent is indicated by a vertical line in the respective plots in Fig. 3b [26–28]. Ex vivo treatment with ibrutinib did not result in inhibition of BTK (pY223), except at the 10,000 nM concentration, which is in agreement with progressive disease on ibrutinib (Fig. 3b, left). However, idelalisib treatment resulted in inhibition of AKT (pS473) (Fig. 3b, middle), and venetoclax treatment induced cleavage of caspase-3 (Fig. 3b, right), suggesting that these therapies were active in the patient’s CLL cells.

Following ibrutinib therapy, the patient received treatment with idelalisib (150 mg b.i.d) (Fig. 3a). Unfortunately, the patient experienced adverse events, and the therapy had to be stopped after four months (Fig. 3a). The schedule was next changed to venetoclax monotherapy (Fig. 3a). The disease responded very well to the treatment, but progressed after ten months. There was no indication of Richter’s transformation.

Since all therapies that were approved for CLL in Norway at this point had been exhausted, we wished to analyze the drug sensitivities of the CLL cells to help guide the next line of therapy for the patient. A blood sample was collected when the patient’s disease was progressing on venetoclax (T1, Fig. 3a), and we performed a drug sensitivity screen on her PBMCs. As a comparison, the same screen was performed on PBMCs from T0, with a custom drug library consisting of 94 single agents and 87 drug combinations at 5 concentrations (1–10,000 nM; 0.1–1000 nM for copanlisib and dasatinib). The tested drugs were either approved for CLL, under clinical investigation in CLL, previously reported to be effective against CLL cells, or derivatives of such. The drug combinations were designed using the fixed molar concentration series identical to those used for single agents. Prior to drug sensitivity testing, the patient-derived cells were co-cultured with irradiated fibroblasts stably expressing APRIL, BAFF, and CD40L for 24 h to prevent spontaneous apoptosis of the CLL cells.

We observed that the sensitivity to most of the single agents (Fig. 3c, left) and drug combinations (Fig. 3c, right) was varying between T0 and T1. The drug sensitivity score (DSS) to venetoclax, an in vitro drug responsiveness metric calculated based on the area under the concentration-response curve [29], was reduced from a high value of 53.7 to 10.6, while the DSS to idelalisib remained unchanged and low (15.5 vs 15.5). The drugs with the highest DSS at both time-points included proteasome inhibitors (bortezomib and ixazomib citrate) and the Bcl-2 antagonist BGB-11417 (Fig. 3c, left). The latter is interesting as it targets the same protein as venetoclax. Both our finding (Fig. 2j, k) and that of others [30] have shown that the pharmacokinetics of BGB-11417 is different from venetoclax. This distinction suggests that BGB-11417 could have additional indications for patients with venetoclax-resistant CLL. To explore this possibility, a clinical trial on BGB-11417 is planned for CLL patients who have previously been treated with venetoclax [30]. Results will provide clinical insight into the efficacy of BGB-1147 in venetoclax-resistant CLL.

The sensitivity to drug combinations was generally reduced from T0 to T1, in particular for those combinations that showed a high DSS at T0 (Fig. 3c, right). The DSS for idelalisib + venetoclax dropped from 53.8 to 25.6 from T0 to T1. However, the sensitivity to other Bcl-2 antagonist combinations remained high at T1, as observed for the copanlisib (PI3K inhibitor) + BGB-11417 and cobimetinib (MEK inhibitor) + venetoclax combinations (Fig. 3c, right).

Although the sensitivity to idelalisib + venetoclax was lower at T1 than at T0, treatment with this combination was attempted following venetoclax therapy since these agents were available in Norway at the time. Idelalisib was administered at 100 mg b.i.d. since 150 mg b.i.d. previously resulted in severe adverse effect. The patient obtained a partial response, and the blood values nearly normalized while on treatment (Fig. 3d). However, the patient suffered intolerable gastrointestinal adverse effects, and the treatment was stopped after three months.

This case demonstrates that ex vivo drug testing can indicate treatment sensitivity, but not treatment tolerability. Since PI3Ki are associated with severe toxicity [31, 32], ex vivo sensitivity to a PI3Ki should not be used as a stand-alone indication for treatment. Future efforts in large patient cohorts are needed to identify predictors of both efficacy and tolerability, which should then be validated in prospective clinical trials.

### Evolution of ex vivo drug sensitivity in response to sequential therapies and treatment stop

We next collected additional patient samples from when the patient was on treatment with idelalisib + venetoclax (T2), and after end of therapy (T3, T4) (Fig. 4a). We performed drug sensitivity testing on the samples collected at T2-T4 and observed that there was a continued trend towards decreased drug sensitivity to drug combinations from T0 to T1-T3 (Fig. 4b). However, the drug sensitivity at T4 reversed to a similar level as seen at T0 (Fig. 4b). This suggests that drug sensitivity can recur when the CLL cells are released from therapeutic pressure. The effect was most pronounced for combinations with a Bcl-2 antagonist (Fig. 4b, right cluster).

We next performed protein profiling by phospho flow cytometry in the serial samples. When the disease progressed after venetoclax treatment (T1), the signals (as arcsinh ratio) for Bcl-2 (pS70) increased from 0.28 to 1.97 (704%) relative to T0, Bcl-2 increased from 2.65 to 4.16 (157%), and Mcl-1 increased from 0.38 to 1.92 (505%). The Bcl-xL signal was moderately elevated at T1 relative to T0 (2.99 vs 2.49; 120%). These findings agree with reported resistance mechanisms to venetoclax [9]. The signals for these anti-apoptotic proteins were reduced after treatment stop at T4 relative to T1, but did not reach the same low level as at T0 (Fig. 4c). We further observed that B-cell signaling was reduced in response to idelalisib + venetoclax therapy (T2), but it increased again when the therapy was stopped (T3, T4) (Fig. 4c). Increased phosphorylation of BTK (pY223) and ERK1/2 (pT180/Y182) at T4 (Fig. 4d, e) was mirrored by increased sensitivity to venetoclax combined either with a BTK or MEK inhibitor (Fig. 4f, g). Unfortunately, the patient passed away due to disease progression, and the in vivo efficacy of these alternative combination therapies could not be tested.

## Discussion

We report a time-limited co-culturing assay that supports durable survival of primary CLL cells for at least 13 days, and mimics in vivo drug resistance signals. By applying the protocol to a patient case of relapsed CLL, we confirmed that ex vivo sensitivity to venetoclax correlates with in vivo response to the same treatment. The method also identified treatment vulnerabilities which were used to guide treatment for the patient. These findings suggest that the protocol can facilitate functional precision medicine in CLL.

Drug sensitivity testing has been successfully implemented to guide large-scale treatment decisions in relapsed/refractory acute leukemia and lymphoma, but, to the best of our knowledge, not yet in CLL [5, 6]. In the EXALT trial (NCT03096821), image-based functional precision medicine was tested prospectively for 143 patients with aggressive hematologic malignancies [5]. Fifty-six of the patients (39%) received precision medicine treatment, and 30 of these patients (54%) obtained clinical benefit as defined by the study protocol (more than 1.3-fold prolonged progression-free survival compared with the previous line of therapy) [5]. A second study integrated drug-response data in a functional precision medicine tumor board to identify actionable drugs for patients with acute myeloid leukemia [6]. A total of 186 patients were included in the study. Thirty-seven patients (20%) received precision medicine treatment with a 59% objective response rate [6]. Although not all patients could receive functionally guided precision medicine in these studies, one reason being that therapies were not available, these studies demonstrate that the approach is feasible and effective in hematologic malignancies. The studies also demonstrate that functional precision medicine is a reasonable alternative or addition to genomic precision medicine, which can guide treatment decisions only for a fraction of cancer patients [3].

One challenge with implementing functional precision medicine for CLL has been that CLL cells undergo rapid, spontaneous apoptosis when they are removed from their natural microenvironment. It is therefore instrumental to have a model of the CLL microenvironment which is compatible with high-throughput drug sensitivity screens in order to translate functional testing of CLL cells to the clinic. We show here that our model can be used for this purpose.

The in-depth characterization of ex vivo drug sensitivity and protein profile of the CLL patient case during sequential lines of therapy showcases the evolution of the disease at a molecular level. We showed that ex vivo sensitivity to targeted therapies, measured either at the protein level or as cell viability, associated with in vivo responses to the respective treatments. Of note, the overall ex vivo sensitivity to treatments decreased in response to consecutive lines of therapies, as expected, with the exception of the recurring drug sensitivity to combination therapies observed after end of the last line of therapy. Interestingly, this change in ex vivo sensitivity was not apparent immediately after end of therapy, but was observed at the last sampling time approximately five months later. This suggests that the effect of the treatment extends beyond treatment duration, which is in agreement with a retrospective study of the prolonged effect of idelalisib + rituximab treatment in CLL [33]. Of interest, we also observed that the patient’s disease remained sensitive to combined treatment with idelalisib + venetoclax after sequential treatment with the monotherapies and relapse on venetoclax. In support of this finding, a retrospective study of heavily pretreated CLL patients who had already received treatment with a BTK inhibitor and venetoclax, showed that combined treatment with these agents provided valuable disease control [34]. If our findings can be confirmed in a larger patient cohort, this may suggest that the absence of drug pressure can create therapeutic windows which can be exploited as a treatment strategy in a clinical setting.

Taken together, our findings support the use of functional testing to guide treatment decisions in CLL. The presented model of the CLL tumor microenvironment will enable clinical implementation of functional precision medicine in CLL, which may improve disease outcomes.

## Materials and methods

### Reagents and antibodies

All compounds and drug combinations that were used in this study are listed in Supplementary Table 1 and Supplementary Table 2, respectively. Antibodies against AKT (pS473) (D9E), AKT (pT308) (C31E5E), Bcl-2 (124), Bcl-xL (54H6), Bim (C34C5), cleaved caspase-3 (Asp175) (D3PE), Ki-67 (D3B5), Mcl-1 (D2W9E), p38 MAPK (pT180/Y182) (28B10), p44/42 MAPK (pT202/Y204) (E10), p90RSK (S380) (D5D8), PCNA (D3H8P), S6-ribosomal protein (pS235/S236) (D57.2.2E), SAPK/JNK (pT183/Y185) (G9), SYK (pY525/Y526) (C87C1) and Tyrosine (pY100) were from Cell Signaling Technologies (Leiden, The Netherlands). Antibodies against Bcl-2 (pS70) (N46-467), Btk (pY223)/Itk (pY180) (N35-86), Btk (pY551) & p-Itk (pY511) (24a/BTK (Y551), IgG1 Kappa (MOPC-21), MEK1 (pS298) (J114-64), MEK1 (pS218)/MEK2 (pS222) (024-836), mTOR (pS2448) (O21-404), NF-κB p65 (pS529) (K10-895.12.50), p53 (pS37) (J159-641.79), PLCγ2 (pY759) (K86-689.37), Rb (pS807/S811) (J112-906), STAT1 (pS272) (K51-856), STAT1 (pY701) (14/P), STAT3 (pS727) (49/p-Stat3), STAT3 (pY705) (4/p-STAT3), STAT5 (pY694) (47), STAT6 (pY614) (18/p-Stat6), TBK1 (pS172) (J133-587), and ZAP70/Syk (pY319/Y352) (17 A/P-ZAP70) were from BD Biosciences (San Jose, CA, USA). These antibodies were conjugated to Alexa Fluor 647. PerCP-Cy5.5 conjugated mouse anti-human CD19 antibody (HIB19) was from eBioscience (San Diego, CA, USA). PE-Cy7 conjugated mouse anti-human CD3 antibody (UCHT1) was from BD Biosciences. BAFF (D7I1U) and CD40 ligand (D5J9Y) antibodies were from Cell Signaling Technologies (Leiden, The Netherlands), and anti-APRIL/CD256 was from Fisher Scientific (Waltham, Massachusetts, USA). Goat F(ab’)2 anti-human IgM was from Southern Biotech (Birmingham, AL, USA). BD Phosflow Fix Buffer I, Perm Buffer III and Fixable Viability Stain (575 V) were from BD Biosciences. Alexa Fluor 488, Pacific Blue and Pacific Orange Succinimidyl Esters were from Thermo Fisher Scientific (Waltham, MA, USA).

### Generation of ligand expressing 3T3 fibroblasts

CD40 ligand (CD40L) L-cells were a gift from Jack Banchereau [35]. Fibroblasts overexpressing human A proliferation-inducing ligand (APRIL), B-cell activating factor (BAFF), or a combination of APRIL and CD40L, were generated by lentiviral transduction of murine NIH/3T3 fibroblasts (CRL-1658, American Type Culture Collection) or CD40L L-cells. Briefly, the open reading frame sequences of human APRIL and BAFF were amplified from normal human B cell cDNA and inserted into the NotI and XbaI cloning sites of the vector pHIV-ZsGreen (Addgene plasmid #18121). HEK293T cells were co-transfected with the pHIV-ZsGreen vector and packaging vectors pCMV-VSV.G (Addgene plasmid #8454) and pCMV-d8.2 dvpr (Addgene plasmid #8455) using FuGENE HD transfection reagent (Fugent LLC) to generate lentiviral particles. Virus-containing supernatant was collected after 48 and 72 h, and concentrated using Amicon Ultra-50 spin columns (EMD Millipore, Merck KGaA, Darmstadt, Germany). The 3T3 cells or CD40L L-cells were transduced by 6 h incubation with virus supernatant in the presence of polybrene (800 µg/mL: Santa Cruz Biotechnology, Dallas, TX). Singly transduced clones expressing APRIL (3T3-APRIL-eGFP) and BAFF (3T3-BAFF-eGFP) were sorted using a BD FACSAria III cell sorter (BD Biosciences). Doubly transduced CD40L/APRIL (CD40LA) cells were selected by culture in medium containing 8 µg/mL puromycin (Sigma-Aldrich) and sorted by FACS based on GFP positivity. All fibroblasts were regularly confirmed mycoplasma negative with the MycoAlert Detection Kit (Lonza, Basel, Switzerland).

### Patient material and ethical considerations

Blood samples from CLL patients were received from the Department of Haematology, Oslo University Hospital, Norway. All participants signed a written informed consent prior to sample collection. The study was approved by the Regional Committee for Medical and Health Research Ethics of South-East Norway (2016/947 and 28507). Research on blood samples was carried out in agreement with the Declaration of Helsinki. Isolation of peripheral blood mononuclear cells (PBMCs) from the CLL samples was performed as previously described [36]. Isolated cells were cryopreserved in liquid nitrogen, which does not affect the functionality of B cells [25]. The characteristics of the patients included in this study can be found in Supplementary Table 3.

### CellTiter-Glo luminescent cell viability assay

Concentration-response experiments were performed as previously described [37]. Briefly, each compound or combination (Supplementary Tables 1, 2) were printed into four 384-well cell culture microplates using the Echo 550 liquid handler (Labcyte Inc., San Jose, CA, USA). Each compound was tested at five different concentrations in ten-fold increments ranging from 1–10,000 nM (0.1–1000 nM for copanlisib and dasatinib). Combinations were designed using the fixed molar concentration series identical to those used for single agents. CLL cells were first co-cultured with irradiated (125 Gy/125 Gy/50 Gy, respectively) GFP-APRIL, GFP-BAFF and GFP-APRIL + CD40L expressing fibroblasts (ratio 1:1:1) for 24 h to mimic the tumor microenvironment and to prevent induction of spontaneous apoptosis. The CLL cells were then separated from the adherent fibroblast layer by carefully re-suspending the culturing medium and transferring it to a separate tube. A single-cell suspension was distributed to each well of the 384-well assay plate using the CERTUS Flex liquid dispenser (Fritz Gyger, Thun, Switzerland). The cells were incubated with the compounds at 37 °C for 72 h. Cell viability was measured using the CellTiter-Glo luminescent cell viability assay (Promega, Madison, WI, USA) following the manufacturer’s instructions. Luminescence was recorded with an EnVision 2102 Multilabel Reader (PerkinElmer, Waltham, MA, USA). The response readout was normalized to the negative (0.1% DMSO) and positive (100 μM benzethonium chloride) controls. The measured concentration-response data were processed with the KNIME software (KNIME AG, Zurich, Switzerland). Calculation of z-prime was used as quality assessment of the experiments. The z-prime was ≥ 0.5 for all experiments. One drug sensitivity screen was performed per patient sample, as supported by the previously demonstrated high reproducibility of the assay [38].

### Phospho flow with fluorescent cell barcoding

Experiments were performed as previously described [39, 40]. Briefly, CLL cells were co-cultured with 3T3 wild-type cells, GFP-APRIL and GFP-APRIL + CD40L expressing fibroblasts, or GFP-APRIL, GFP-BAFF and GFP-APRIL + CD40L expressing fibroblasts for 24 h. The CLL cells were then separated from the fibroblasts, stained with fixable viability stain, fixed and permeabilized. The cells were stored in the permeabilization buffer at −80 °C until further processing. After thawing, the cells were washed with PBS supplemented with 2% FBS, and stained with the surface marker anti-CD19 and antibodies against intracellular proteins. The samples were analyzed with a BD LSR Fortessa cytometer (BD Biosciences) equipped with 488 nm, 561 nm, 640 nm and 407 nm lasers. The data were analyzed in Cytobank (https://cellmass.cytobank.org/cytobank/) as previously described [39].

### Data analyses

Data were processed and visualized using GraphPad Prism 9 (San Diego, CA, USA). Applied statistical tests are indicated in the figure legends. To quantify the compound responses, a modified drug sensitivity score (DSS) was calculated for each sample and compound separately [29]. Area under the concentration-response curve was calculated using an activity window from 100% to 10%, and a window from the minimum concentration tested to the concentration where the viability reached 10%. DSS3 metric was used, without the division by the logarithm of the upper asymptote of the logistic curve. A higher DSS indicates higher sensitivity to the treatment. Illustrations were made with BioRender.com.

## Supplementary information

Supplementary Table 1

Supplementery Table 2

Supplementary Table 3

Supplementary figure legends

Supplementary Figure 1

## Data Availability

The data generated in this study are available upon reasonable request to the corresponding author (sigrid.skanland@ous-research.no).
