# Supplementary material for: A tumor microenvironment model of chronic lymphocytic leukemia enables drug sensitivity testing to guide precision medicine
Source: Cell Death Discov. 2023 Apr 13;9:125. doi: 10.1038/s41420-023-01426-w (PMC10101987; doi:10.1038/s41420-023-01426-w)
Supplement: Supplementary file 2 — Supplementery Table 2 [file 41420_2023_1426_MOESM2_ESM.docx]

**Supplementary Table 2.** Drug combinations

| **Drug 1** | **Drug 2** |
| --- | --- |
| 2-chlorodeoxyadenosine | Valproic acid |
| Acalabrutinib | AZD6738 |
| Acalabrutinib | BGB-10188 |
| Acalabrutinib | BGB-11417 |
| Acalabrutinib | Buparlisib |
| Acalabrutinib | Compound 7n |
| Acalabrutinib | Copanlisib |
| Acalabrutinib | Duvelisib |
| Acalabrutinib | Idelalisib |
| Acalabrutinib | Nemiralisib |
| Acalabrutinib | Pictilisib |
| Acalabrutinib | Pilaralisib |
| Acalabrutinib | Quizartinib |
| Acalabrutinib | Umbralisib |
| Acalabrutinib | Venetoclax |
| Acalabrutinib | ZSTK474 |
| Alisertib | Crizotinib |
| BGB-10188 | BGB-11417 |
| BGB-10188 | Ibrutinib |
| BGB-10188 | Venetoclax |
| BGB-10188 | Zanubrutinib |
| BGB-11417 | Copanlisib |
| BGB-11417 | Duvelisib |
| BGB-11417 | Ibrutinib |
| BGB-11417 | Idelalisib |
| BGB-11417 | Zanubrutinib |
| Binimetinib | Venetoclax |
| Buparlisib | Ibrutinib |
| Buparlisib | Venetoclax |
| Cabozantinib | Ruxolitinib |
| Chlorambucil | Ibrutinib |
| Chlorambucil | Lenalidomide |
| Cobimetinib | Venetoclax |
| Compound 7n | Ibrutinib |
| Compound 7n | Venetoclax |
| Copanlisib | Ibrutinib |
| Copanlisib | Venetoclax |
| Copanlisib | Zanubrutinib |
| Cytarabine | Nutlin 3a |
| Dasatinib | Venetoclax |
| Dexamethasone | Lenalidomide |
| Doramapimod | Palbociclib |
| Duvelisib | Ibrutinib |
| Duvelisib | Venetoclax |
| Duvelisib | Zanubrutinib |
| Fludarabine | Ibrutinib |
| Ibrutinib | Idelalisib |
| Ibrutinib | Nemiralisib |
| Ibrutinib | Pictilisib |
| Ibrutinib | Pilaralisib |
| Ibrutinib | Quizartinib |
| Ibrutinib | Selinexor |
| Ibrutinib | SNX-5422 |
| Ibrutinib | Umbralisib |
| Ibrutinib | Venetoclax |
| Ibrutinib | ZSTK474 |
| Idelalisib | JQ1 |
| Idelalisib | Quizartinib |
| Idelalisib | Ruxolitinib |
| Idelalisib | Trametinib |
| Idelalisib | Venetoclax |
| Idelalisib | Zanubrutinib |
| JQ1 | Palbociclib |
| JQ1 | Ruxolitinib |
| JQ1 | Sorafenib |
| Lenalidomide | Methylprednisolone |
| Nemiralisib | Venetoclax |
| Palbociclib | Quizartinib |
| Palbociclib | Ruxolitinib |
| Palbociclib | Sorafenib |
| Palbociclib | Trametinib |
| Palbociclib | Venetoclax |
| Panobinostat | Ruxolitinib |
| PD0325901 | Venetoclax |
| Pictilisib | Venetoclax |
| Pilaralisib | Venetoclax |
| Pimasertib | Venetoclax |
| Refametinib | Venetoclax |
| Ruxolitinib | Venetoclax |
| Selumetinib | Venetoclax |
| Sorafenib | Venetoclax |
| Trametinib | Venetoclax |
| U0126 | Venetoclax |
| Umbralisib | Venetoclax |
| Vandetanib | Vemurafenib |
| Venetoclax | Zanubrutinib |
| Venetoclax | ZSTK474 |
