# Supplementary material for: A tumor microenvironment model of chronic lymphocytic leukemia enables drug sensitivity testing to guide precision medicine
Source: Cell Death Discov. 2023 Apr 13;9:125. doi: 10.1038/s41420-023-01426-w (PMC10101987; doi:10.1038/s41420-023-01426-w)
Supplement: Supplementary file 3 — Supplementary Table 3 [file 41420_2023_1426_MOESM3_ESM.docx]

| **Patient ID** | **Gender** | **Age** | **IGHV usage** | **Homology with germline** | **Treatment prior to procurement** |
| --- | --- | --- | --- | --- | --- |
| CLL116 | M | 82 | 4-59 | 93,4% | None |
| CLL161 | M | 55 | 4-34/1-3 | 91%/81% | None |
| CLL177 | F | 69 | 4-59 | 94,5% | None |
| CLL187 | M | 66 | 1-69 | 93,2% | None |
| CLL206 | F | 65 | 4-39 | 98,7% | None |
| CLL227 | F | 69 | 1-2 | 93% | None |
| CLL248 | M | 73 | 4-34 | 97,5% | FCR |
| CLL249 | F | 80 | n.e. | NA | None |
| CLL250 | F | 82 | 3-23 | 99,7% | BR |
| CLL251 | F | 63 | 3-48*02 | 96,2% | None |

**Supplementary Table 3.** Patient characteristics

BR, bendamustine/rituximab; F, female; FCR, fludarabine/cyclophosphamide/rituximab; M, male; NA, not applicable; n.e., not evaluable
