# Supplementary material for: A tumor microenvironment model of chronic lymphocytic leukemia enables drug sensitivity testing to guide precision medicine
Source: Cell Death Discov. 2023 Apr 13;9:125. doi: 10.1038/s41420-023-01426-w (PMC10101987; doi:10.1038/s41420-023-01426-w)
Supplement: Supplementary file 4 — Supplementary figure legends [file 41420_2023_1426_MOESM4_ESM.docx]

**SUPPLEMENTARY FIGURE LEGENDS**

**Supplementary Figure 1. Cell viability and proliferation of CLL cells in response to CD40L/APRIL/BAFF stimulation**

**a.** Peripheral blood mononuclear cells (PBMCs) from CLL patient samples were co-cultured at a 10:1 ratio with irradiated wild-type 3T3 fibroblasts or GFP-APRIL, GFP-BAFF and GFP-APRIL + CD40L fibroblasts for 24h. The CLL cells were then separated from the adherent fibroblast layer and kept in culture in the incubator for 6 days. One million cells were washed and stained with fixable viability stain and fixed at days 0, 3, and 6. The cells were analyzed by flow cytometry. Dot plots showing one representative experiment and fraction of live cells are gated. The graph shows percentage of live cells for day 0, day 3, and day 6 after co-culture (mean ± SEM, n=3). Statistics were performed with a two-tailed unpaired t-test.

**b.** PBMCs from CLL patient samples were either fixed at baseline (B) or after co-culture with the different fibroblast lines for 24h as described in a). The cells were then permeabilized and stained with the indicated antibodies. The cells were analyzed by flow cytometry. The first 3 bars show 3T3 control co-cultured CLL cells. The 3 bars to the right show CD40L/APRIL/BAFF co-cultured CLL cells. The CLL cells were analyzed as percent positive counts relative to the baseline (mean ± SEM, n=3). The graphs show measurements for baseline (B), day 3, and day 6 after co-culture. Statistics were performed with a two-tailed unpaired t-test. *p<0.05.
