# Supplementary figures and images for: A tumor microenvironment model of chronic lymphocytic leukemia enables drug sensitivity testing to guide precision medicine
Source: Cell Death Discov. 2023 Apr 13;9:125. doi: 10.1038/s41420-023-01426-w (PMC10101987; doi:10.1038/s41420-023-01426-w)

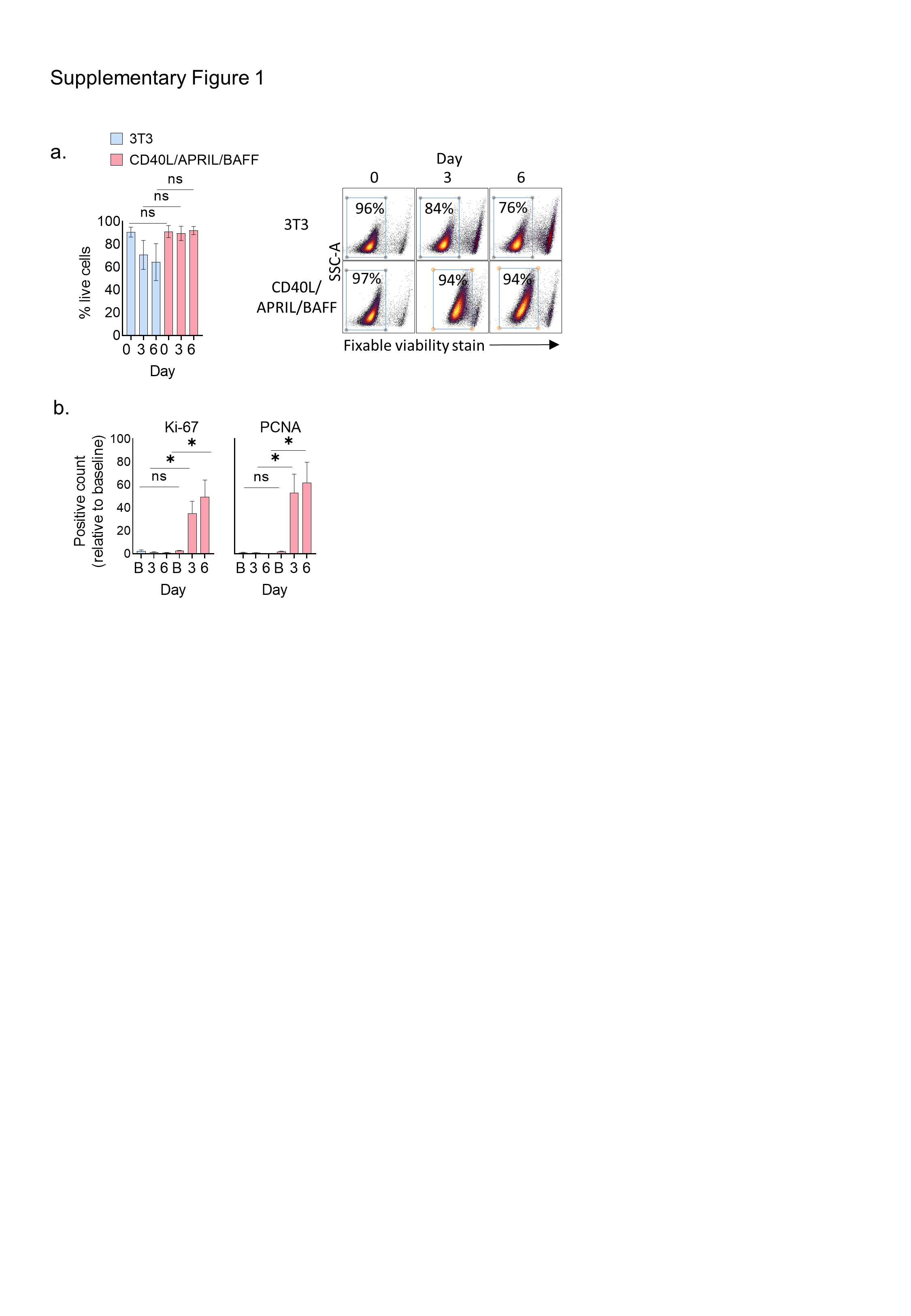

Supplement: Supplementary file 5 — Supplementary Figure 1 [file 41420_2023_1426_MOESM5_ESM.tif]
